# Laboratory test of Single Landmark registration method for ultrasound-based navigation in laparoscopy using an open-source platform

**DOI:** 10.1007/s11548-018-1830-7

**Published:** 2018-08-03

**Authors:** Javier Pérez de Frutos, Erlend F. Hofstad, Ole Vegard Solberg, Geir Arne Tangen, Frank Lindseth, Thomas Langø, Ole Jakob Elle, Ronald Mårvik

**Affiliations:** 1 Department of Health, SINTEF A.S., Trondheim, Norway; 2 Computer Science Department, NTNU, Trondheim, Norway; 3 The Intervention Centre, Oslo Rikhospital, Oslo, Norway; 4 Department of Gastrointestinal Surgery, St. Olavs Hospital, Trondheim, Norway

**Keywords:** Registration, Laparoscopy, Computed-assisted surgery, Ultrasound, Multimodal visualization

## Abstract

**Purpose:**

Test the feasibility of the novel Single Landmark image-to-patient registration method for use in the operating room for future clinical trials. The algorithm is implemented in the open-source platform CustusX, a computer-aided intervention research platform dedicated to intraoperative navigation and ultrasound, with an interface for laparoscopic ultrasound probes.

**Methods:**

The Single Landmark method is compared to fiducial landmark on an IOUSFAN (Kyoto Kagaku Co., Ltd., Japan) soft tissue abdominal phantom and T2 magnetic resonance scans of it.

**Results:**

The experiments show that the accuracy of the Single Landmark registration is good close to the registered point, increasing with the distance from this point (12.4 mm error at 60 mm away from the registered point). In this point, the registration accuracy is mainly dominated by the accuracy of the user when clicking on the ultrasound image. In the presented set-up, the time required to perform the Single Landmark registration is 40% less than for the FLRM.

**Conclusion:**

The Single Landmark registration is suitable for being integrated in a laparoscopic workflow. The statistical analysis shows robustness against translational displacements of the patient and improvements in terms of time. The proposed method allows the clinician to accurately register lesions intraoperatively by clicking on these in the ultrasound image provided by the ultrasound transducer. The Single Landmark registration method can be further combined with other more accurate registration approaches improving the registration at relevant points defined by the clinicians.

## Introduction and background

With the improvements in minimally invasive surgery techniques and instruments in recent years, there is a trend towards more use of the laparoscopic approach, although open surgery remains the gold standard for abdominal surgeries. Advantages of laparoscopic surgery include a less traumatizing intervention and a better post-operative phase for the patient, also decreased morbidity, quicker recovery, less blood loss and improved long-term outcomes when compared to open surgery [1–4]. Nonetheless, there are concerns like risk of gas embolism due to pneumoperitoneum [2] or the limited space and field of view. To overcome the reduced field of view, the surgeons make use of a laparoscopic video camera for instrument guidance and other image modalities like ultrasound (US) for inspection and assessment of the lesion.

Laparoscopic ultrasound (LUS) was introduced originally by Yamakawa and co-workers in 1958 [5], providing real-time information of the inside of the organs. Jakimowicz and Reuers introduced LUS scanning for examination of the biliary tree during laparoscopic cholecystectomy in 1991 [6]. Since then, the use of LUS has expanded with the increase in minimally invasive procedures. Today, LUS is applied in a large number of procedures, such as screening for lymph nodes identification and tumour scanning; diagnostic detection, localization and assessment of the extent of a tumour; and in therapeutic procedures as a guidance tool [7, 8].

With the technical improvements in image processing, computers and tracking systems, Image-Guided Navigation Platforms (IGNPs) emerged as an assisting tool for laparoscopic surgery. This software platform allows the surgeon to plan the operation beforehand [9] and also to have accurate and relevant information about the anatomy of the patient during surgery, with three-dimensional (3D) models of the anatomy and the used tools in the same view [10]. The combination of navigation and LUS will enable more soft tissue surgery in the abdomen to be performed with the laparoscopic technique. Tracked LUS together with registered preoperative data, e.g. computed tomography (CT), magnetic resonance imaging (MRI) or positron emission tomography (PET), provides a real-time US view matched to segmented models from preoperative data. This gives the surgeon an updated map of the target anatomy and structures during the procedure [11]. Navigated LUS also makes easier to relate the oblique two-dimensional (2D) US images to relevant anatomy.

Surgical margins are a major concern in hepatectomy interventions like hepatocellular carcinoma (HCC) and colorectal liver metastases (CLM) resections. The recommended surgical margin is of 2 cm for HCC and 1 cm for CLM [12]. IGNP can possibly enable surgeons to perform successful interventions with smaller resection margins, through the combination of medical images and intraoperative registration.

Image-to-patient registration is the first requirement to perform navigated LUS. This is spatially locating the preoperative images and the patient with respect to a common coordinate reference frame. For this purpose, tracking systems detect and compute the position and orientation of the tools and the patient in the operating room (OR), creating a virtual environment with a common coordinate reference frame. After completing the registration, the image information can be overlaid and shown together with the real-time position of the tools and the patient, allowing further navigation. Currently, there are four spatial tracking technologies being used in the OR: mechanical, optical, electromagnetic and acoustic [8, 13].

In this study, an optical tracking system was used to locate the tools and the liver phantom. Optical tracking systems typically consist of highly reflective markers or infrared emitting diodes attached to the patient (or OR table) and tools, infrared (IR) light sources to illuminate the reflective markers, IR cameras to detect the markers or diodes, and software that computes the position and orientation, i.e. tracking six spatial degrees of freedom, of the objects based on the spatial location of the markers.

**Fig. 1:**
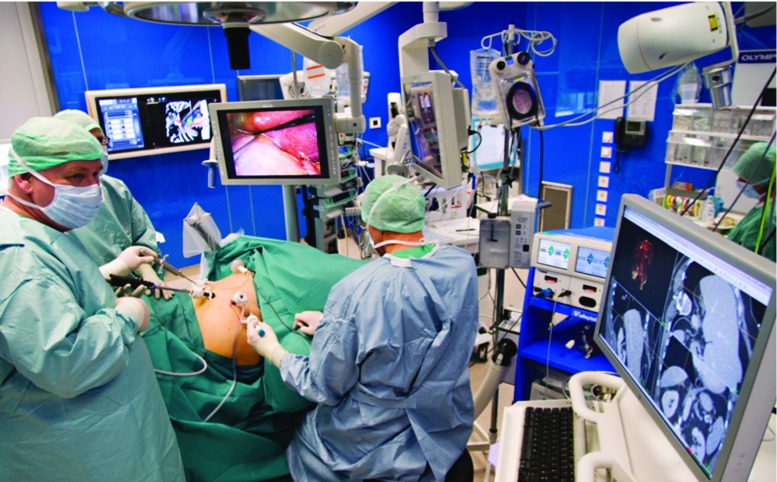
Navigation in laparoscopic surgery based on preoperative CT images

Figure 1 shows the setting from a laparoscopic adrenalectomy using preoperative 3D CT images for the initial in-the-OR planning of the procedure, just before inserting the trocars. The view direction of the volume was then set by the view direction of the laparoscope as it was introduced. The LUS image could be displayed in the same scene, with an indication of the probe position using the open-source CustusX [9] platform.

This article presents the Single Landmark registration method (SLRM), as part of the open-source platform CustusX [9] for US-navigated laparoscopic surgery. This software allows the surgeon to integrate and fuse real-time LUS images with preoperative data, segmented models from medical images, and models of tracked tools. The main novelty of this study is a fast and simple to use method for image-to-patient registration in the OR, validated on a soft tissue abdominal US compatible phantom. This is an incremental step to reach navigated LUS integrated in the clinical workflow. The ultimate goal is to efficiently combine all information sources to provide a real-time visualization of the anatomy of the patient combined with the tracked instruments.

## Materials and methods

### CustusX image-guided intervention platform

CustusX is an open-source IGNP developed by the research group at the Norwegian Centre for Innovative Ultrasound Solutions in Trondheim, Norway [9]. This platform integrates medical image visualization and real-time tracking of the surgical instruments, providing complete navigation for surgery in minimal invasive procedures. It also includes an interface to acquire real-time US images, which can be overlaid onto the virtual model of the patient.

The software is based on C++ and uses the Qt framework [14]. CustusX uses several external open-source libraries like VTK [15] for image visualization and processing, ITK [16] for segmentation and registration algorithms, and CTK [17] for processing DICOM files.

### Single Landmark registration

The SLRM is a rigid image-to-patient registration algorithm that uses the orientation of a tracked tool and an anatomical reference point or landmark, for aligning the image data to the reference frame of the patient. The registration involves two phases: an initial registration using the orientation of the tool and a landmark that enables navigation, and the re-registration using the target lesion(s) intraoperatively. Although a surgical pointer is used here, the orientation and reference points can be acquired with any tracked instrument, as suggested in [11].

**Fig. 2:**
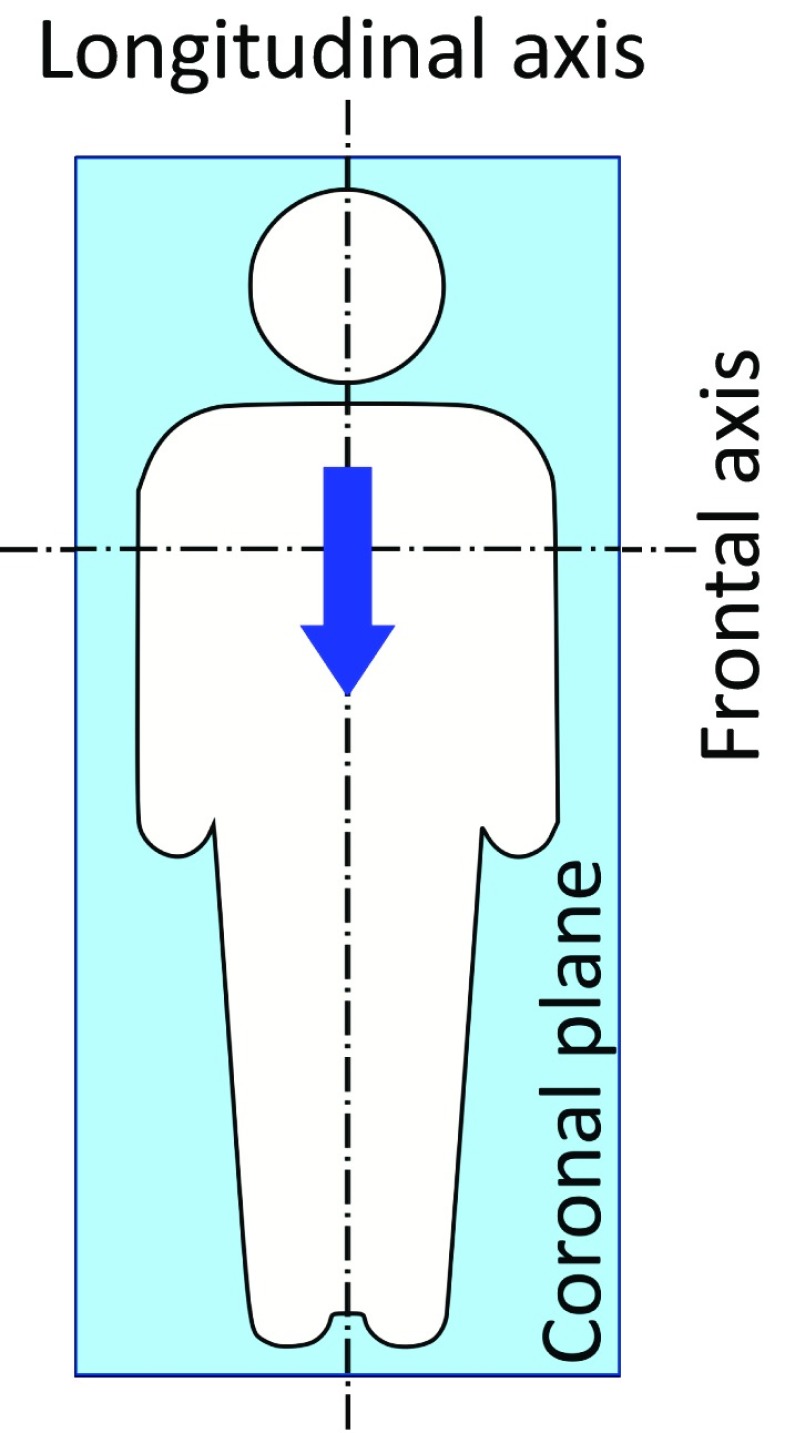
Suggested location and orientation of the tracked tool (arrow), in this case, over the sternum of the patient

The algorithm assumes the tracked tool is oriented along the longitudinal axis of the patient and lying parallel to the coronal plane as suggested in Fig. 2. Because, in the prone orientation, the pointer will face downwards and might occlude the reflective markers, SLRM allows to specify whether the patient is in supine or prone position, so the pointer can be oriented upwards in both situations. Incorrect orientation of the tracked tool would result in misalignments between the virtual model and the patient anatomy reference frame. Therefore, the user is allowed to sample the orientation several times.

**Fig. 3:**
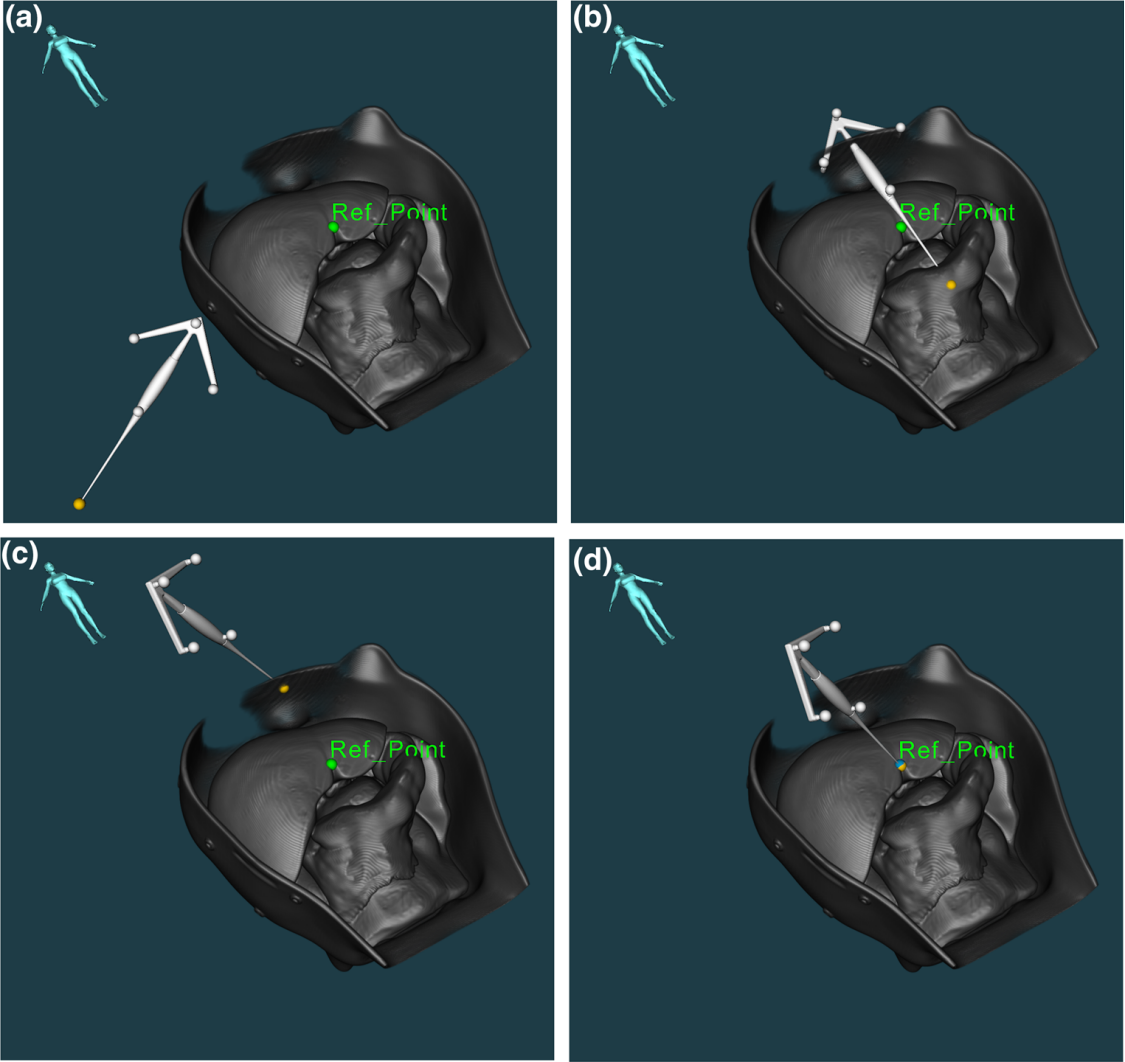
SLRM image-to-patient registration steps: **a** initial location of tool as to sample the orientation; **b** the virtual model is oriented accordingly to the acquired orientation; **c** the reference point is marked with the pointer on the phantom; and **d** complete registration of the virtual model after manually sampling the reference point

Reference points must be first manually marked in the preoperative data images, to perform the registration. By registering a reference point in both the patient and the 3D volume, the virtual model is rigidly translated (see Fig. 3a–d). Thus, there is an accurate match between the virtual model and anatomy in that point.

For the second phase of the method, new landmarks can be sampled during surgery, like tumours or anatomical structures. These new points can be used to re-register the patient model, improving the accuracy of the initial registration in a close neighbourhood of the point. Whenever a new point is registered, the transformation offset is updated to match such point but keeping the orientation constant. Therefore, the full potential of the SLRM can then be exploited using a LUS transducer, as it is the main tool used by surgeons to confirm the location of the lesions intraoperatively. Once the tumour is visible in the US image, the user can register the virtual volume including the lesion by clicking on the centre of the tumour shown in the US image. The platform allows to zoom in the US slice, improving the point sampling of the user and minimizing the effect of the screen resolution.

**Fig. 4:**
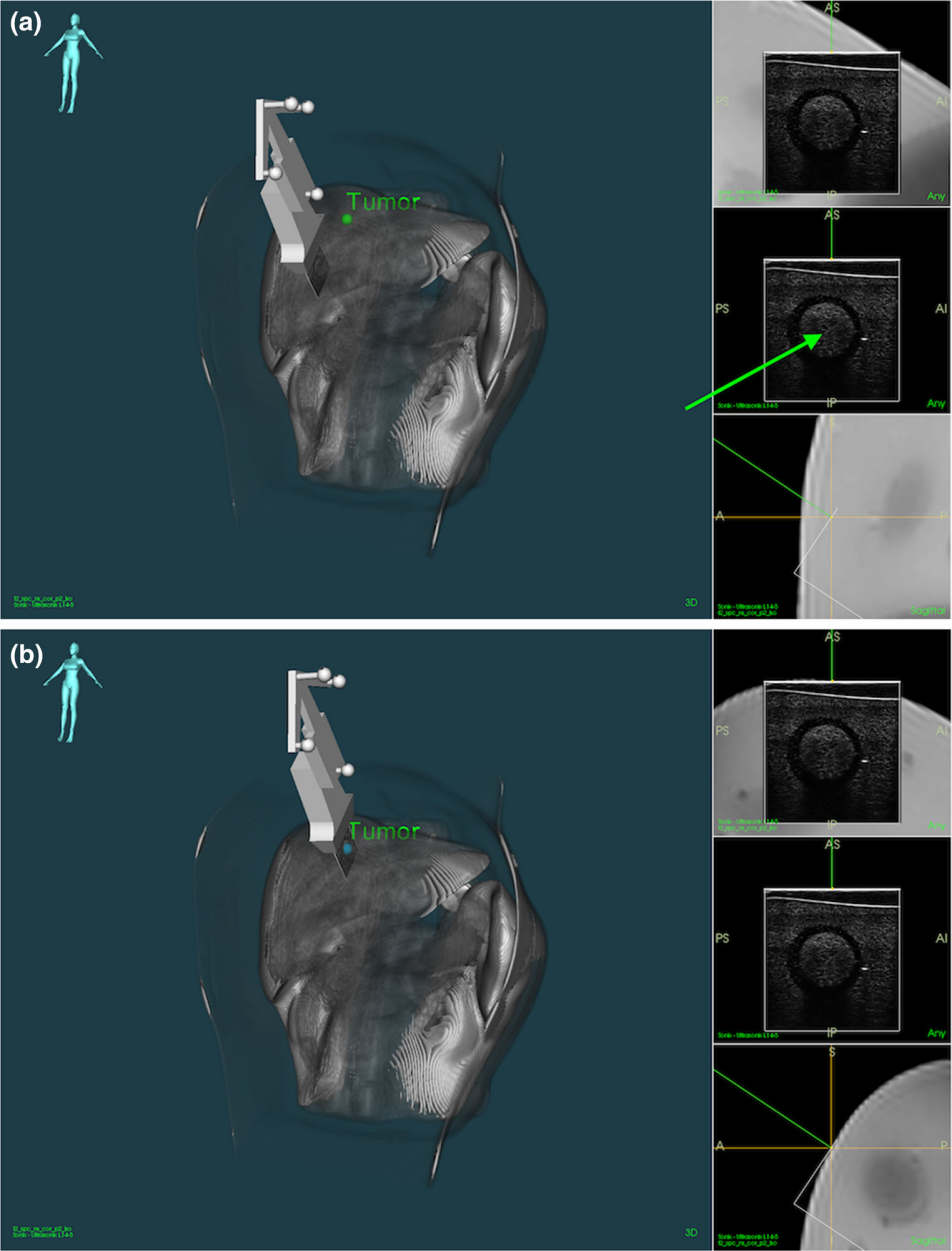
SLRM registration of a lesion (green point) using the US image. **a** Before and **b** after the registration

Figure 4a, b shows the procedure to register a lesion using the US image. The same virtual model as the one displayed in Fig. 3 is rendered translucent so the tumour (green point) can be seen. In Fig. 4a, the tumour shown corresponds to the US image on the right side. After clicking on the centre of the lesion in any of the US images (green arrow), the SLRM registers the selected tumour with the point clicked by the user.

Optical tracking has been used in this study. However, in a real situation surgical tools like the LUS would be electromagnetically tracked, as the optical tracking systems require line of sight and are not able to track the movements of the articulated tip once within the abdomen.

As aforementioned, the SLRM applies a rigid transformation on the virtual model. Therefore, anatomical movements like respiratory motion, or pneumoperitoneum, are not taken into account. These factors could result in deformations on the liver of several centimetres [18]. However, due to the local registration using the LUS, the effect of these deformations can be reduced locally on the registered lesion.

### Abdominal Intraoperative and Laparoscopic Ultrasound Phantom IOUSFAN

**Fig. 5:**
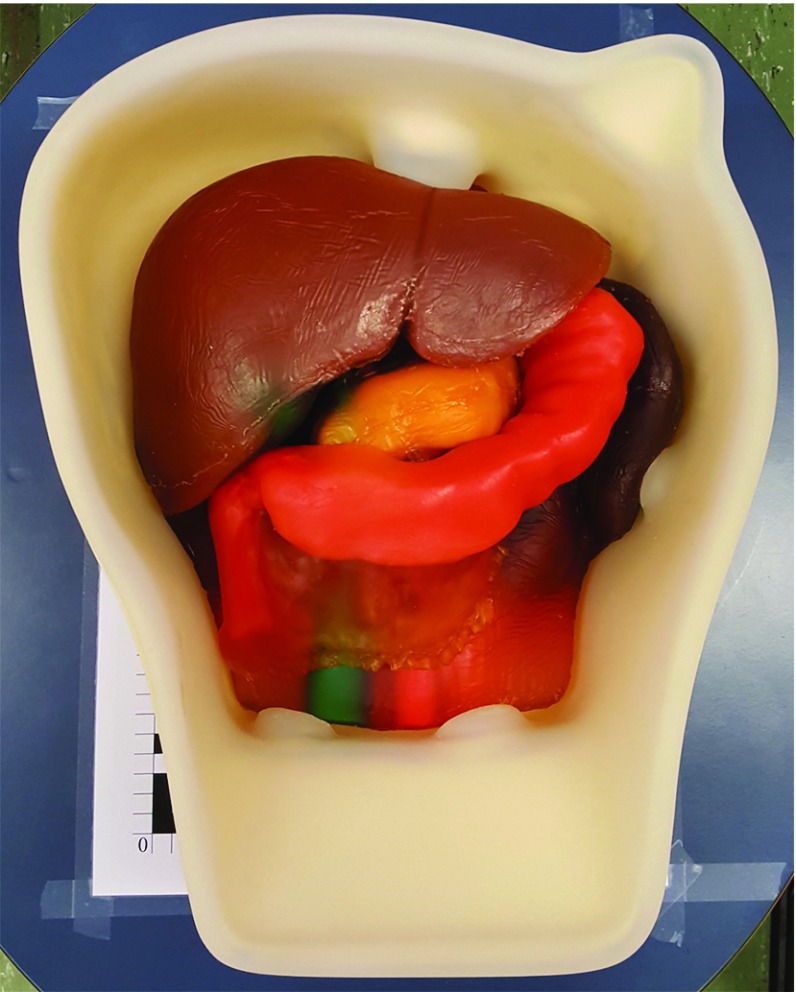
Abdominal Intraoperative and Laparoscopic Ultrasound Phantom IOUSFAN [19]

For this experiment, an Abdominal Intraoperative and Laparoscopic Ultrasound Phantom IOUSFAN (Kyoto Kagaku Co., Ltd., Japan) [19] was used (see Fig. 5). The phantom contains the most relevant abdominal structures and includes different types of lesions within each of them. The whole phantom is contained in a rigid case, where fiducial reference markers were attached before acquiring MR and CT scans.

### Preoperative data

The MR T2 DICOM data were imported in the navigation system, though the same results could be obtained using other image modalities. A 3D reconstruction of the IOUSFAN phantom is shown in Figs. 3 and 4.

### Navigation equipment

**Fig. 6:**
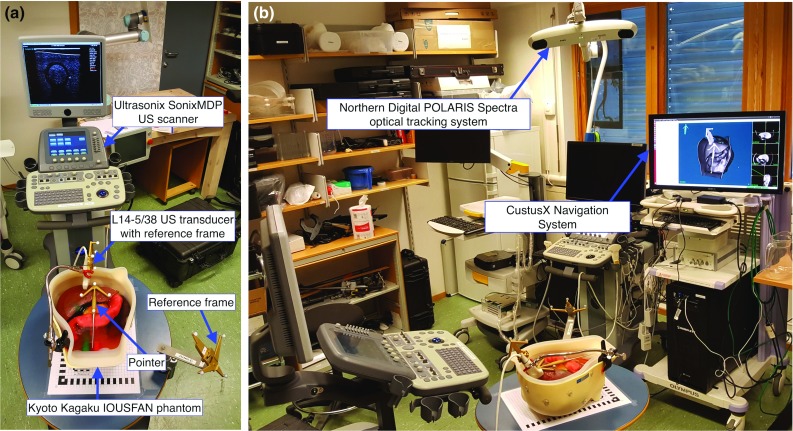
**a** SonixMDP US scanner, IOUSFAN and tools and frames with optical markers; **b** POLARIS optical tracking system and CustusX navigation system

US images were acquired with a SonixMDP US scanner (Ultrasonix Medical Corp., Richmond, Canada) and a L14-5/38 linear transducer (Prosonic Gyeongbuk, South Korea), as seen in Fig. 6a. A tracking frame with reflective markers was attached to the US probe and the table, becoming the latter the OR reference frame. A grid was fixed to the table with a resolution of centimetres, to measure the displacements of the phantom. A surgical pointer with reflective markers was used to register the landmarks. The POLARIS Spectra^®^ (Northern Digital^®^ Inc., Canada) and NDI^®^ spherical passive retro-reflective markers were used for optical tracking [20]. Figure 6a and b shows the experiment set-up.

The US probe spatial calibration was verified using the evaluation wire phantom used in [21] and the Wire Widget of CustusX [9]. The verification process compares the centreline of 3D US reconstructions of the wires with the calibrated virtual model, where the crossing point of the wires is used as the calibration point. Different trajectories were followed when scanning, acquiring between 100 and 200 US images per scan. The surgical pointer was calibrated using the pivot calibration option available in the Tool Tracker^®^ application of NDI ToolBox^®^ (20 s scan, 60 Hz), resulting in an average of 604 valid samples per scan. The average calibration errors are shown in Table 1. Both calibrations were done using POLARIS Spectra^®^ for tracking.

**Table 1:** Calibration errors in millimetres of the US probe and the surgical pointer

Instrument	US probe	Surgical pointer
Average	0.21	0.44
Standard deviation	0.49	0.05

### Set-up

**Fig. 7:**
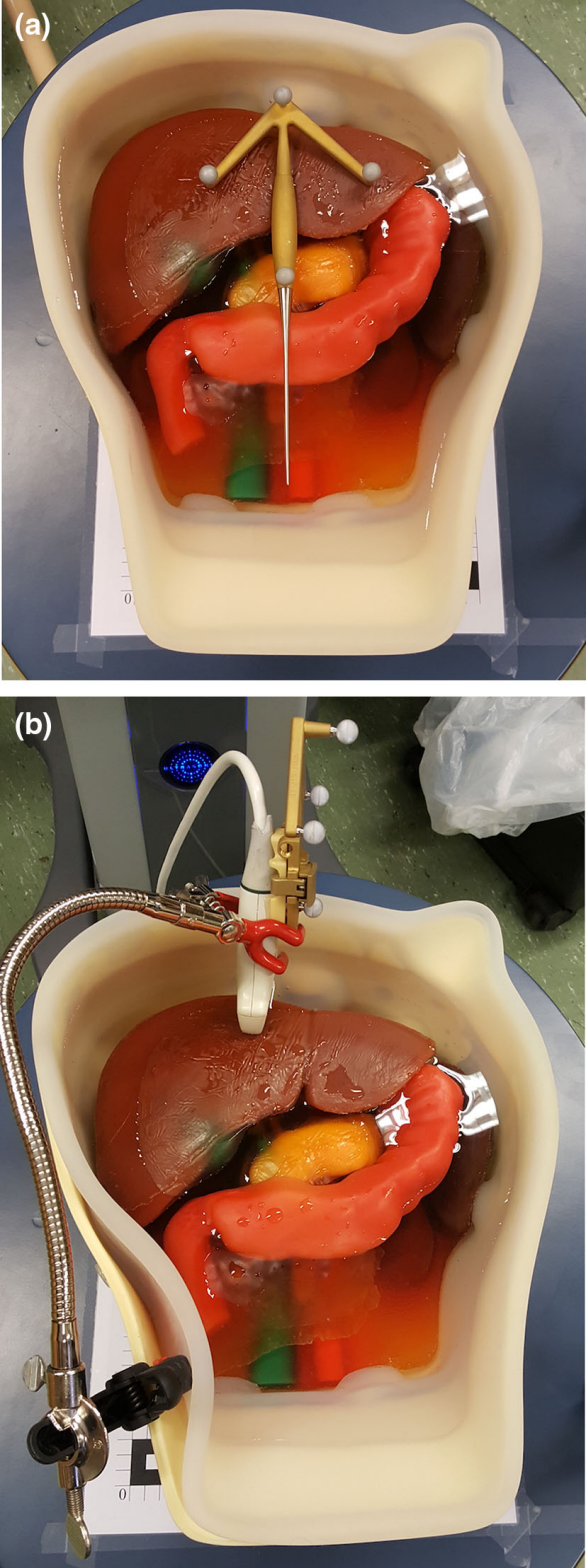
**a** Optical pointer placed on the phantom to perform the image-to-patient SLRM registration. **b** US probe attached to the phantom

The IOUSFAN phantom liver is placed on the table, and the US probe is attached to the case so the same lesion is used for each displacement and sample (see Fig. 7b) and oriented to obtain a clear image of the lesion. Using the POLARIS Spectra^®^ optical tracking system and the reference frame attached to the table, the US probe and the pointer are tracked and spatially located (see Fig. 6). The Ultrasonix scanner streams US images to CustusX through an OpenIGTLink [22] network.

Fiducial points are manually marked in the virtual model using CustusX. The image-to-patient registration is accomplished using the surgical pointer to sample the landmarks and the orientation of the phantom.

### Experiment protocol

The aim of this experiment was to test the feasibility of the SLRM to be used in future clinical trials. For this, SLRM was compared to fiducial-based rigid landmark registration method (FLRM). For both methods, the phantom is moved ten times by 10, 50 and 100 mm in the frontal and longitudinal axes, independently. After each displacement, the virtual model is re-registered to correct the displacement using the same reference points as in the first image-to-patient registration. The tumour location, tracked with the US, is then used for verification. The centre of the tumour is manually marked using the US image and compared to the position of the same tumour manually marked in the virtual model. The chosen lesion is located 60 mm away from the registered point.

The initial image-to-patient SLRM is performed by leaving the pointer over the liver and oriented as in Fig. 2 (see Fig. 7a). Then, the reference point is registered using the pointer. Because of its easy access, the reference point chosen for this experiment is where the round and the falciform ligament meet on the liver (*Ref_Point* in Fig. 3a, d). This same reference point is used for the re-registration after each displacement. Nonetheless, as long as the fiducial point can be located in both the virtual model and the patient anatomy, users are free to choose any more accessible fiducial point.

For comparison, a FLRM is done using five fiducial markers distributed over the case of the phantom. The same procedure is followed as with the SLRM, performing a complete registration after each displacement and tracking the location of the tumour using the US transducer.

User time, i.e., time required by the user to perform an image-to-patient registration, is measured for each registration performed using a chronometer. For the SLRM, the time measured corresponds to that between the moment the orientation of the tool is recorded and when the reference point is registered. In the case of the FLRM, the user time is measured between the sampling of the first and the fifth fiducial points.

## Experimental results

**Fig. 8:**
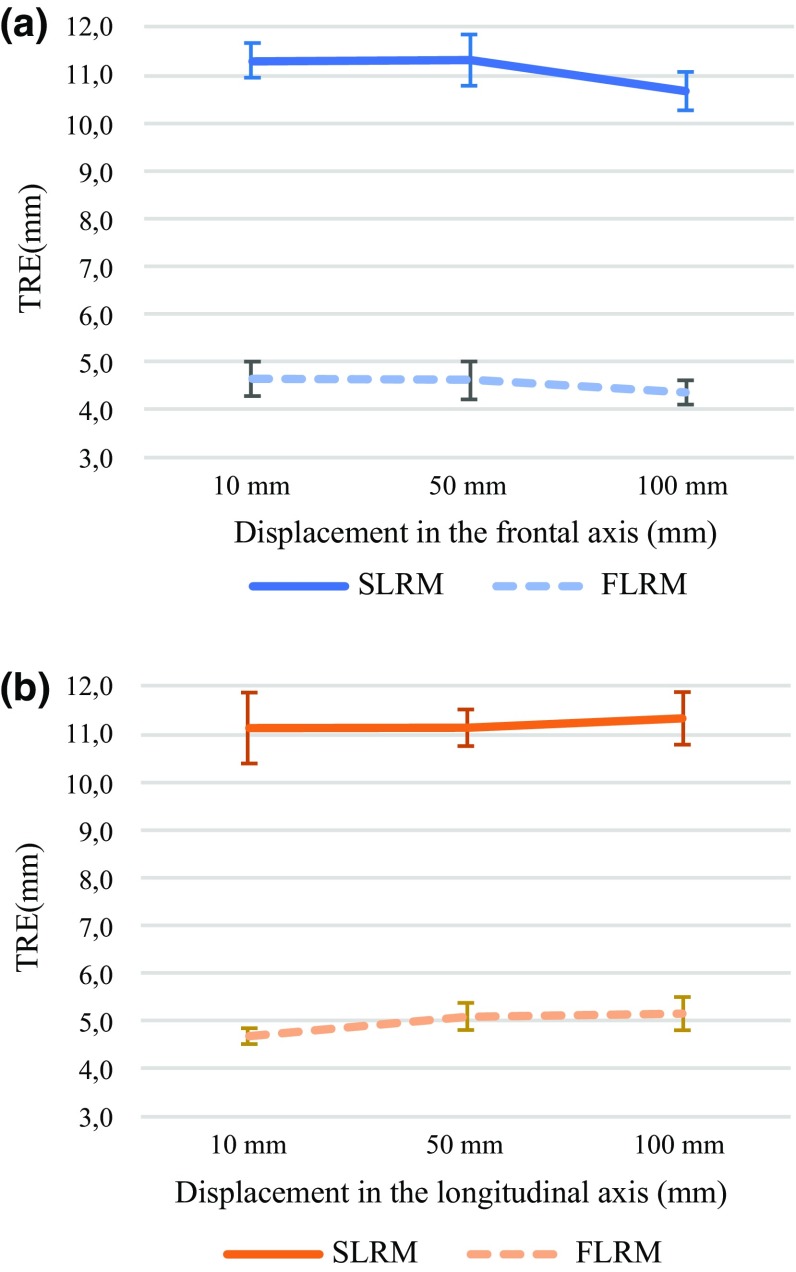
TRE values shown in Table 2 for the SLRM and FLRM in the **a** frontal axis and the **b** longitudinal axis

A total of 60 target registration error (TRE) [23] samples were computed for each registration method. The TRE is measured as the Euclidean distance between the centre of the tumour, found using the US probe, and the location of this same lesion in the virtual model, for each displacement in the frontal and longitudinal axes (see Experiment protocol). Table 2 shows the average TRE results. These same values are plotted in Fig. 8a and b where it can be seen that the average TRE does not vary greatly with the direction the distance displaced. The repeatability of each group is computed as the standard deviation of the mean.

**Table 2:** TRE between the tumour visualized the US image and in the MRI scan, using SLRM and FLRM

Displacement	10 mm		50 mm		100 mm	
Displacement axis	Frontal	Longitudinal	Frontal	Longitudinal	Frontal	Longitudinal
SLRM						
Average	11.3	11.1	11.3	11.1	10.7	11.3
Standard deviation	0.4	0.7	0.5	0.4	0.4	0.5
Minimum	10.5	10.1	10.3	10.6	10.1	10.4
Maximum	11.8	12.4	11.9	12.0	11.3	12.0
Repeatability	0.11	0.23	0.17	0.12	0.13	0.17
FLRM						
Average	4.6	4.7	4.6	5.1	4.4	5.2
Standard deviation	0.2	0.4	0.3	0.4	0.3	0.3
Minimum	4.4	4.3	4.2	4.7	3.7	4.7
Maximum	5.0	5.5	5.1	5.8	4.9	5.6
Repeatability	0.05	0.09	0.11	0.11	0.13	0.08

The data were analysed using IBM^®^ SPSS^®^ Statistics Version 25 software [24]. A *p* value below 0.05 was considered statistically significant. SLRM TRE data were analysed using a one-way ANOVA. Variables were categorized in six groups according to the displacement and the axis, i.e. X10, X50, X100, Y10, Y50 and Y100. Levene’s test showed no difference between the variances of the groups [*F*(5, 54) = 2.226, *p* = 0.065]. The ANOVA deemed statistical difference between the means [*F*(5) = 2.437, *p* = 0.046]. However, multiple composition analysis using Tukey’s honest significant difference and Scheffe’s method showed no statistical difference between pairs of groups, with a significance level of 95%. Therefore, the means of the groups are statistically similar, and thus, SLM is robust against displacements of the patient.

A one-way ANOVA was used to compare the TRE for the two registration methods. The samples were grouped according to the method, i.e. SLRM or FLRM. Variances of the groups were found to be statistically different [*F*(1, 118) = 4.34, *p* = 0.039]. Therefore, a Welch test of equal means was performed, resulting in statistically dissimilar means [*F*(1, 113.397) = 5004.32, *p* = 0.000]. So, the TRE performance of SLRM and FLRM is statistically different, FLRM showing the best results.

A total of 60 samples of time were measured for each method. Statistics are shown in Table 3. The time samples were classified in SLRM and FLRM and analysed using a T-test analysis. Statistical differences were found between the variances of the groups [*F*(1, 118) = 29.994, *p* = 0.000], and the test proved the means between the groups to be different [*t*(75.292) = 51.627, *p* = 0.000]. Therefore, there is a significant difference in time required between the two methods, SLRM being faster than FLRM (40% for the current experiment).

**Table 3:** User time in seconds for the SLRM and FLRM

Registration method	FLRM	SLRM
Average	19.63	7.62
Standard deviation	1.68	0.63
Repeatability	0.22	0.08

## Discussion

In this study, the novel SLRM image-to-patient intraoperative registration method is introduced and validated for clinical use. It is currently implemented in CustusX [9] IGNP. The experiments tested the capability of the algorithm to perform intraoperative image-to-patient registration, with special focus on the complexity of the steps and the required user time. Experimental set-up comprised of an L14-5/38 linear transducer connected to an UltraSonix SonixMDP scanner, a surgical pointer, a POLARIS Spectra^®^ optical tracker, and an IOUSFAN soft tissue abdominal phantom. Tool calibration was conducted as described in navigation equipment section. Because the US probe is fixed to the phantom case, only the spatial calibration was verified.

During the experiment, the surgical pointer is used for setting the orientation, speeding the initial registration process. However, it is possible to perform a conventional patient registration, by using several fiducials or contour registration, and then keep the resulting orientation of the virtual model instead of using a pointer. This may result in a more correct orientation, improving accuracy during the re-registration phase in a larger area around the registered point.

The multiple composition statistical analysis of the TRE shows no correlation between the SLRM TRE performance and the displacement of the phantom. The reported repeatability values are considered adequate for the presented study. It is also shown that the TRE is highly correlated to the registration method, i.e. SLRM or FLRM, FLRM showing the best results. The user time shows a statistical difference between methods, being the SLRM faster than the FLRM (see Table 3).

Therefore, it is concluded that the SLRM image-to-patient registration is suitable to be integrated in a laparoscopic intervention workflow in combination with a tracked LUS. The use of SLRM will result in a major improvement in terms of time consumption without compromising the TRE close to the registered point. The strategy is to use this simple and efficient method as a starting point for an intraoperative fine-tuning registration method based on 3D US data acquired by the tracked 2D LUS probe, while staging the liver in the initial phase before resection.

However, it must be considered that the algorithm assumes that the user provides the correct orientation of the patient and the correct location of the reference point. Therefore, the SLRM TRE is highly dependent on the precision with which the user samples these parameters. Discrepancies between the preoperative scans and the position and location of the patient anatomy during the operation, e.g. anatomical shift involving rotation, when the liver is mobilized, will be an additional source of error to be considered. Also, the point-sampling accuracy of the user when clicking on the US slice would affect the outcome of the registration, though this effect is mitigated with the possibility of zoom in the US image to better aim for the desired point. Further experimentation would be required to quantify the effect of these error sources; however, this is out of the scope of the current study. Human accuracy with laparoscopic tools and computer input devices have been studied in [25–27].

To improve the accuracy at different regions, the surgeon is able to re-register new points of such areas that can be visualized in the LUS images. The method matches the virtual model with the updated location of the intraoperatively registered point. Therefore, the proposed method allows the surgeon to accurately register lesions, especially in situations where FLRM cannot be performed, e.g. due to large differences between the preoperative data and the situation in the OR.

As such, future work will focus on combining the SLRM algorithm with more robust or accurate registration methods, e.g. matching corresponding vessel structures in the virtual model and US, where the SLRM could be beneficial to locally improve the TRE within a close area around the lesion. Furthermore, deformable registration methods could be used to further improve the TRE around the registered point using SLRM.

## Conclusion

This study introduces the novel SLRM using an open-source platform for US-based navigation in laparoscopy. SLRM has been shown suitable to be integrated in the normal workflow of a laparoscopic surgical procedure. Furthermore, the accuracy of the tracking system, the calibration and the registration are well within the recommended surgical margin limits for hepatectomy interventions [12, 21].

Reduced user time and simple steps are two of the principal advantages of the proposed method, together with the possibility of performing the image-to-patient registration during the preparation of the patient and intraoperatively using the LUS. Thus, providing real-time and accurate information of the anatomy of the organ to the surgeon in the neighbourhood of the registered point, where other registration methods may not be applicable.

In the current implementation, the SLRM performs rigid transformations over the image data. Thus, the algorithm is sensitive to factors such as the point-sampling accuracy of the user when clicking on the centre of the lesion.

Therefore, future work will focus on integrating SLRM with more robust registration techniques. Furthermore, SLRM can be combined with deformable registration algorithms to improve accuracy in real time in the region around the lesion.
